# Supplementary material for: PDGFA-associated protein 1 protects mature B lymphocytes from stress-induced cell death and promotes antibody gene diversification
Source: J Exp Med. 2020 Jul 1;217(10):e20200137. doi: 10.1084/jem.20200137 (PMC7537392; doi:10.1084/jem.20200137)
Supplement: Table S2 — lists the oligonucleotides used in this study. [file JEM_20200137_TableS2.docx]

Table S2. List of oligonucleotides used in this study

| Name | Sequence (5′ to 3′) | Reference |
| --- | --- | --- |
| Pdap1F allele genotyping (PCR primer) |  |  |
| MDV_p313 (Fw) | GTCCTATGATTGGACCAAC | This paper |
| MDV_p226 (Rev) | GGGAAGGCAGGCTGTATGTT | This paper |
| MDV_p223 (Fw) | CTGGGGAAAAGGAGGCTCTG | This paper |
| **CRISPR-Cas9 gene targeting (gRNA)** | | |
| g*Pdap1-1* | AGTGAGGCAGTATACGAGCC | This paper |
| g*Pdap1-2* | CATTGAGAACCCCAACCGCG | This paper |
| g*Pdap1-3* | ACTGGATCTGGACGGGCCAA | This paper |
| g*Pdap1-4* | GAAGGTCACGCAACTGGATC | This paper |
| g*Pdap1-5* | TTGTCTGTGCCACGCGGTTG | This paper |
| g*Pdap1-6* | GTTGTCTGTGCCACGCGGTT | This paper |
| g*53bp1* | GAGTGTACGGACTTCTCGAA | (Delgado-Benito et al., 2018) |
| g*Random-1* | GCGAGGTATTCGGCTCCGCG | (Delgado-Benito et al., 2018) |
| g*Random-2* | ATGTTGCAGTTCGGCTCGAT | (Delgado-Benito et al., 2018) |
| g*Random-3* | ACGTGTAAGGCGAACGCCTT | This paper |
| **Quantitative PCR** |  |  |
| ***Aicda*** | | |
| AID-F (Fw) | GAAAGTCACGCTGGAGACCG | (Xu et al., 2015) |
| AID-R (Rev) | TCTCATGCCGTCCCTTGG | (Xu et al., 2015) |
| 119-AID (Fw) | GAGGGAGTCAAGAAAGTCACGCTGGA | (Muramatsu et al., 2000) |
| 118-AID (Rev) | GGCTGAGGTTAGGGTTCCATCTCAG | (Muramatsu et al., 2000) |
| MDV_p504 (Rev) | AGGGTGGCAAGCAGACGAGG | This paper |
| **Postspliced *GLT*** | | |
| ImF (Fw) | CTCTGGCCCTGCTTATTGTTG | (Muramatsu et al., 2000) |
| CmR (Rev) | GAAGACATTTGGGAAGGACTGACT | (Muramatsu et al., 2000) |
| **Postspliced *GLTγ1*** | | |
| Ig1 (Fw) | GGCCCTTCCAGATCTTTGAG | (Muramatsu et al., 2000) |
| Cg1R (Rev) | GGATCCAGAGTTCCAGGTCACT | (Muramatsu et al., 2000) |
| **Postspliced *GLTγ3*** | | |
| Ig3F (Fw) | TGGGCAAGTGGATCTGAACA | (Muramatsu et al., 2000) |
| Cg3R (Rev) | CTCAGGGAAGTAGCCTTTGACA | (Muramatsu et al., 2000) |
| **Postspliced *GLTγ2b*** | | |
| MDV_p243 (Fw) | CACTGGGCCTTTCCAGAACTA | (Muramatsu et al., 2000) |
| MDV_p244 (Rev) | CACTGAGCTGCTCATAGTGTAGAGTC | (Muramatsu et al., 2000) |
| **Postspliced *GLTα*** | | |
| IaF (Fw) | CCTGGCTGTTCCCCTATGAA | (Muramatsu et al., 2000) |
| CaR (Rev) | GAGCTGGTGGGAGTGTCAGTG | (Muramatsu et al., 2000) |
| **Housekeeping** | | |
| Ubc_Fw | GCCCAGTGTTACCACCAAGA | (Albershardt et al., 2012) |
| Ubc_Rev | CCCATCACACCCAAGAACA | (Albershardt et al., 2012) |
| Gapdh_Fw | TGTGTCCGTCGTGGATCTGA | (Albershardt et al., 2012) |
| Gapdh_Rev | TTGCTGTTGAAGTCGCAGGAG | (Albershardt et al., 2012) |
| **Electrophoretic analysis of Xbp1 splicing (PCR primer)** | | |
| Xbp1_Fw | AAACAGAGTAGCAGCGCAGACTGC | (Madaro et al., 2013) |
| Xbp1_Rev | TCCTTCTGGGTAGACCTCTGGGAG | (Madaro et al., 2013) |
| **SHM analysis** | | |
| ***J_H_4*** | | |
| VHA (Fw) | ARGCCTGGGRCTTCAGTGAAG | (Sander et al., 2015) |
| VHE (Fw) | GTGGAGTCTGGGGGAGGCTTA | (Sander et al., 2015) |
| JH4_intron (Rev) | CTCCACCAGACCTCTCTAGACAGC | (Sander et al., 2015) |
| ***J_K_5*** | | |
| VK (Fw) | GGCTGCAGSTTCAGTGGCAGTGGRTCWGGRAC | (Rouaud et al., 2013) |
| JK5_PR (Rev) | AGCGAATTCAACTTAGGAGACAAAAGAGAGAAC | (Rouaud et al., 2013) |
| **pMX-Pdap1-3XFlag cloning (PCR primer)** | | |
| MDV_p137 (Fw) | ACGCGGATCCATGCCTAAAGGAGGAAGAAAGG | This paper |
| MDV_p138 (Rev) | GATGTCATGATCTTTATAATCACCGTCATGGTCTTTGTAGTCTCCACCCTTATTCAGGGAGAGTGACTG | This paper |
| MDV_p73 (Rev) | TTTTCCTTTTGCGGCCGCCTCGAGTCACTTGTCATCGTCATCCTTGTAATCGATGTCATGATCTTTATAATCACC | This paper |
